# Linguistic and Psychometric Validation of the Cognition Bolt-On Version of the Japanese EQ-5D-5L for the Elderly

**DOI:** 10.3233/JAD-221080

**Published:** 2023-02-14

**Authors:** Ataru Igarashi, Yukinori Sakata, Mie Azuma-Kasai, Harue Kamiyama, Mika Kawaguchi, Kiyoyuki Tomita, Mika Ishii, Manabu Ikeda

**Affiliations:** a Department of Public Health, Yokohama City University School of Medicine, Kanagawa, Japan; b Department of Health Economics and Outcomes Research, Graduate School of Pharmaceutical Sciences, The University of Tokyo, Tokyo, Japan; c Eisai Co., Ltd. Tokyo, Japan; d Life Co., Ltd., Tokyo Japan; e Clinical Study Support, Inc., Aichi, Japan; f Department of Psychiatry, Osaka University Graduate School of Medicine, Osaka, Japan

**Keywords:** Cognition, health status, quality of life, psychometrics, public health

## Abstract

**Background:**

The need for a cognition bolt-on version of the EQ-5D, which would capture cognitive impairment by adding a dimension to the generic instrument assessing health status, has been increasing in Japan.

**Objective:**

To develop a cognition bolt-on version of the 5-level EQ-5D (EQ-5D-5L+C), we linguistically validated a cognition dimension and psychometrically validated the EQ-5D-5L+C.

**Methods:**

Following linguistic validation of the cognition dimension, psychometric validation of the EQ-5D-5L+C proxy version utilized anonymized data collected from nursing home residents between October 2021 to April 2022. The validity, reliability, and sensitivity to change were evaluated.

**Results:**

Data from 254 participants, including the finalized Japanese EQ-5D-5L+C proxy version, were analyzed for the psychometric validation. Mean (±standard deviation) age and Mini-Mental State Examination (MMSE) scores were 87.14±7.29 years and 15.76±8.46, respectively. The correlation was strongest between the cognition dimension and MMSE scores (*r*_s_ = –0.640). Test-retest reliability was good in the cognition dimension in both baseline and two-time points (3 months: *k* = 0.644; 6 months: *k* = 0.656). Although a correlation between changes in the cognition dimension and those in the MMSE score from baseline was weak (3 months: *r*_s_ = –0.191; 6 months: *r*_s_ = –0.267), a correlation with changes in the MMSE score was higher when the cognition dimension was added compared to the EQ-5D alone (3 months: *r*_s_ = –0.142 versus *r*_s_ = –0.074).

**Conclusion:**

The Japanese EQ-5D-5L+C proxy version developed is a valid tool that captures health status including cognitive function, with a consideration for an over-time assessment. The benefits in adding the cognition dimension to the EQ-5D-5L to assess health state were suggested.

## INTRODUCTION

The EQ-5D is a commonly used generic instrument developed by the EuroQol Group (Rotterdam, Netherlands) to assess health-related quality of life (HRQOL) and has been utilized for health technology assessment (HTA) worldwide. It evaluates five dimensions including mobility, self-care, usual activities, pain/discomfort, and anxiety/depression [1, 2].

Over the past decades, there have been discussions among researchers on the needs for condition-specific “bolt-on” versions of the EQ-5D. This would extend the standard five dimensions by adding a single or multiple dimensions to the EQ-5D to better capture disability caused by various conditions, such as cognition [3– 6], vision, hearing, tiredness [7], sleep [8], and psoriasis [9]. Dementia and cognitive impairment, which people often experience with aging, have been one of the areas focused. Krabbe et al. demonstrated that an addition of a cognitive dimension to the EQ-5D improves its ability to capture the health status of people [4]. Subsequently, Arons et al. suggested considering the cognition bolt-on for dementia given that it contributed capturing the impaired cognitive function over time compared to the EQ-5D [5]. The cognition bolt-on has been further studied by Janssen et al. with a 5-level response (EQ-5D-5L) [6], and a significant impact of the addition of a cognition dimension to the EQ-5D from a valuation perspective was reported.

Japan is currently facing an advanced aging society. In 2020, the population aged 65 years or older in Japan accounted for 28.7% of the overall Japanese population [10]. The proportion is projected to increase further up to 35.3% by 2040. Moreover, the prevalence of dementia among the Japanese elderly population is reported to be 16.7% in 2020, with a projection of 24.5% in 2060 [11]. With this large proportion of people aged 65 years or older and expected increase in the prevalence of dementia in this population in mind, an instrument which can evaluate HRQOL including cognitive function will be of benefit. However, no previous research has been conducted for a cognition bolt-on to the Japanese EQ-5D.

In this study, to capture health status in individuals with cognitive impairment among the Japanese elderly, we aimed to develop the cognition bolt-on version of the EQ-5D-5L (hereafter, the EQ-5D-5L+C). This is the cognition bolt-on version of the Japanese EQ-5D-5L by adding a cognition dimension with reference to the bolt-on version previously published by Janssen et al. [6]. To achieve this, linguistic validation was conducted for the added cognition dimension, followed by the psychometric validation of the Japanese EQ-5D-5L+C.

## MATERIALS AND METHODS

### Study design

This study consisted of two phases: 1) adding the cognition dimension to the Japanese EQ-5D-5L with reference to the bolt-on version previously published by Janssen et al. [6] and validating the cognition dimension linguistically and 2) psychometrically validating the cognition bolt-on version of the EQ-5D-5L (i.e., EQ-5D-5L+C). The modification to the EQ-5D-5L was conducted under the permission granted by the EuroQol Group (Rotterdam, the Netherlands). This study was conducted as a part of the study entitled “Efficiency and Optimization of Care in Nursing Homes from the Perspectives of QOL and Medical Economics”, approved by the ethics committee of the Graduate School of Pharmaceutical Science, Faculty of Pharmaceutical Science, The University of Tokyo. The study was conducted following the local Ethical Guidelines for Medical and Biological Research Involving Human Subjects.

### Linguistic validation

#### Forward translation

After obtaining the permission from the EuroQol Group, the English cognition dimension was obtained from the original developer of the cognition dimension (M. F. Janssen), and it was cross-culturally adapted for the Japanese language in accordance with previously published guidelines [12, 13]. Two independent translators translated the cognitive dimension to Japanese and reconciled the two translated versions into one. One of the authors (MI), medical expert, reviewed the draft, and it was revised where needed.

#### Back translation

Another Japanese-English bilingual translator who was blinded to the original English version translated the Japanese draft back into English. The original developer of the cognition dimension reviewed the back translation whether it was conceptually equivalent to the original cognition dimension. Following his confirmation, a provisional Japanese cognition dimension was finalized.

#### Pilot test

The pilot test was conducted in July 2021. Five Japanese-speaking individuals aged 20 years or older who worked as a caregiver at a nursing home or who were the main caregiver of his/her family member with dementia at home were recruited and completed the provisional Japanese cognition dimension added to the Japanese EQ-5D-5L proxy version after their consent to participate in the pilot test. Following its completion, the participants were debriefed online regarding cognition dimension and the overall impression of the EQ-5D-5L+C, to ensure that the cognitive dimension was understood and answerable. The summarized debriefing results were reviewed. The recruitment and debriefing following an interview guide were conducted by INTAGE Healthcare Inc. (Tokyo, Japan).

#### Finalization of the Japanese version

After the pilot test, the provisional Japanese cognition dimension was to be revised where needed and was finalized after the authors’ agreements. The Japanese cognition dimension embedded in the Japanese EQ-5D-5L (EQ-5D-5L+C) proxy version was finalized.

### Psychometric validation

#### Participants

The finalized Japanese EQ-5D-5L+C proxy version was administered in all the nursing home residents of six nursing homes associated with Life Group, Inc. (Tokyo, Japan). The collected anonymous processed data were used for psychometric validation of the Japanese EQ-5D-5L+C. Consent was obtained from the nursing home residents or his/her potential surrogate (e.g., family member and adult guardian) using the informed consent form approved by the ethics committee. Residents with data of the EQ-5D-5L+C, EQ-5D-5L [2, 6, 14– 17], and Mini-Mental State Examination (MMSE) [18] available at least two time points of month 0 and month 3 during the study period were included in the analysis. No exclusion criteria were applied.

The target sample size was a total of 250 individuals which was considered plausible to collect data at those participating six nursing homes.

#### Data source

Data originally collected every three months between October 2021 and April 2022 were extracted. Data were originally collected via questionnaires that were responded by proxy, such as facility managers, care managers, nurses, or other caregiving staff at nursing homes at 0 month, 3 months, and 6 months from October 2021(baseline).

The data source included demographics and clinical characteristics of participants, including age, sex, independence in activity of daily living owing to dementia assessed using the “Criteria for determination of the daily life independence level of the elderly with dementia” [19], prescribed anti-dementia drugs (i.e., donepezil, galantamine, rivastigmine, memantine, and yokukansan/YiganSan), activities of daily living (ADL) assessed using Barthel Index (BI) [20], occupation/position of the proxies who responded to the EQ-5D-5L+C and EQ-5D-5L [2, 6, 14– 17], EQ-5D-5L+C (proxy version responded by proxy), EQ-5D-5L (proxy version responded by proxy), and MMSE [18].

### Questionnaire data

#### Criteria for determination of the daily life independence level of the elderly with dementia

The independence in activities of daily living in relation to the presence and symptoms of dementia was assessed using the criteria for determination of the daily life independence level of the elderly with dementia. The criteria are commonly used to assist in identifying individuals eligible for long-term care under the insurance system of Japan. This tool was originally developed to enable healthcare professionals to objectively and quickly determine the level of independence in daily living (i.e., level of care required) for elderly people diagnosed with dementia by a physician [19]. Ranks are categorized as I, II, II a, II b, III, III a, III b, IV, and M. The higher rank indicates the presence of more severe symptoms that interfere with daily life and the more frequent difficulties in behavior and communication.

#### BI

Assessment of ADL was performed by BI, which measures functional independence in the domains of personal care and mobility such as feeding, moving from wheelchair to bed and return, grooming, transferring to and from a toilet, bathing, walking on a level surface, going up and down stairs, dressing, and continence of bowels and bladder on a 10-item scale [20]. The score ranges from 0 to 100, the lower score indicating higher degree of one’s dependence on assistance from others.

#### EQ-5D-5L (proxy version)

The EQ-5D-5L was used to evaluate HRQOL. The EQ-5D-5L has a descriptive profile of the five dimensions and the EQ Visual Analogue scale (EQ-VAS). Each dimension has five-level responses, i.e., no, slight, moderate, severe, and unable to/extreme problems [2, 14, 15]. The EQ-5D index score ranges from 0 (death) to 1 (full health), calculated based on value sets developed for the Japanese population [16, 17]. The EQ-VAS, a person’s self-perceived health on a vertical VAS, provides a single score ranging from 0 (worst imaginable health) to 100 (best imaginable health).

#### MMSE

To assess cognitive function and severity of dementia, the MMSE was used. The MMSE is an 11-item questionnaire which covers five areas of cognitive function (i.e., orientation, registration, attention and calculation, recall, and language) [18]. The maximum score is 30, and lower score indicates higher severity of dementia. The MMSE score is categorized as follows: 30, no cognitive impairment; 26– 29, mild cognitive impairment; 21– 25, mild dementia; 11– 20, moderate dementia; and 0– 10, severe dementia with reference to a previous report [21].

### Statistical analysis

Demographic and clinical characteristics of the participants at baseline were descriptively analyzed. Ceiling and floor effects in each dimension of the EQ-5D-5L+C at baseline, 3 months, and 6 months were analyzed, calculating the percentage of the participants scoring the best and worst status. Above 70% was considered to be adverse [22].

Construct validity was analyzed to evaluate whether the instrument measures the construct of interest. Spearman’s rank order correlation coefficients between each EQ-5D-5L+C dimensions and MMSE scores at baseline were analyzed. A correlation coefficient was interpreted as follows: 0.1, weak correlation; 0.3, moderate correlation; and 0.5, strong correlation [23]. Based on the existing literature, we hypothesized that the MMSE score presents at least moderate correlation coefficients with the self-care and usual activities dimensions and a strong correlation with the cognition dimension [3, 24].

Test-retest reliability was evaluated to ensure that the level of the EQ-5D-5L+C cognition dimension is stable when cognitive function is unchanged. The agreements in the cognition dimension between baseline and 3 months and between baseline and 6 months were evaluated among participants whose MMSE category had not changed between the two time points. The Cohen’s Kappa coefficients were calculated using the Fleiss-Cohen weights, based on an inverse-square spacing [25, 26]. The kappa coefficient was interpreted as follows: poor, <0.20; fair, 0.21– 0.40; moderate, 0.41– 0.60; good, 0.61– 0.80; and very good, 0.81– 1.00 [27].

Sensitivity to change in the EQ-5D-5L+C cognition dimension was evaluated based on the correlation with changes in the MMSE score between two sets of time points: baseline and 3 months and baseline and 6 months, using Spearman’s rank order correlation coefficients. A correlation coefficient was interpreted in the same manner as construct validity. Additionally, to evaluate whether the addition of the cognition dimension improves the sensitivity to change in the EQ-5D-5L+C, we compared the sensitivity to change in both EQ-5D-5L and EQ-5D-5L+C. As value sets for the EQ-5D-5L+C are not yet developed, a total score of the EQ-5D-5L and EQ-5D-5L+C was each calculated by adding the level of five or six dimensions.

Statistical analyses were performed using SAS release 9.4 (SAS Institute Inc., Cary, NC, USA) and R version 3.4.0 or above (The R Foundation for Statistical Computing, Vienna, Austria). Missing values were not included.

## RESULTS

### Linguistic validation

#### Translation

Regarding the description of the bolt-on dimension “COGNITION (memory, comprehension, concentration, thinking)”, the discussion focused on the translation of “cognition” and “memory”. The “cognition” was translated as “cognitive function” initially at the forward translation stage for easy understanding although neither “cognition” nor “cognitive function” can be familiar in laypersons. Through the discussion with the medical expert, as these Japanese terms, which were not necessarily easy to understand, are interchangeably used even among clinical professionals, this translation was then revised to “cognition” to simply retain the original expression in English. Instead, based on the medical expert’s opinion, the translation of “memory” was additionally supplemented with the expression “forgetfulness”, which Japanese speakers tend to be more familiar with as memory-related symptoms entailed by cognitive impairment and which is even used for as a part of outpatient department name in the hospitals. Thus, the translation was revised as “memory [forgetfulness]”.

In the back translation draft, “cognition” was translated as “cognitive function”. Although the original developer confirmed that there was no problem with this translation as well as the supplemental description of “forgetfulness”, the authors and translators also agreed that “cognitive function” is more appropriate in this context and corresponds to the expression of the status. A provisional Japanese cognition dimension was finalized after “cognition” was revised to “cognitive function” in Japanese.

#### Pilot test

Five formal or informal caregivers responded to the provisional version of the EQ-5D-5L+C without difficulties in understanding and answering. With this, we confirmed that no change was needed for the provisional version of the EQ-5D-5L+C. The final version of the cognition dimension is shown in the Supplementary Figure 1.

### Psychometric validation

Data from 254 nursing home residents were extracted from the database. Of those, data from 235 residents have response data from three time points: baseline, 3 months, and 6 months.

#### Participant’s characteristics

Baseline characteristics of the participants are shown in Table 1. The mean (standard deviation [SD]) age of the participants was 87.14 (7.29) years, and they were predominantly women (68.9%). The mean MMSE score was 15.76 (8.46), with over 85% classified into mild to severe dementia categories. Donepezil was the most frequently prescribed anti-dementia drug in less than 8% of the participants. Proxies who responded for the participants were primarily registered nurses and other nursing home staff other than facility managers or care managers (39.8% and 46.1%, respectively).

**Table 1:** Baseline characteristics of participating nursing home residents (*n* = 254)

Characteristics	*n* (%)
Age (y), Mean±SD	87.14 ±7.29
Sex	
Men	79 (31.1)
Women	175 (68.9)
Criteria for determination of the daily life independence level of the elderly with dementia	
I	9 (3.5)
II	9 (3.5)
Iia	20 (7.9)
IIb	68 (26.8)
III	38 (15.0)
IIIa	41 (16.1)
IIIb	26 (10.2)
IV	23 (9.1)
M	4 (1.6)
Not applicable	15 (5.9)
Missing	1 (0.4)
Occupations/position of proxy	
Facility manager	14 (5.5)
Care manager	21 (8.3)
Registered nurse	101 (39.8)
Other staff	117 (46.1)
EQ-5D-5L	
Mobility	
No problems	44 (17.3)
Slight problems	55 (21.7)
Moderate problems	33 (13.0)
Severe problems	28 (11.0)
Unable	93 (36.6)
Missing	1 (0.4)
Self-care	
No problems	57 (22.4)
Slight problems	58 (22.8)
Moderate problems	47 (18.5)
Severe problems	36 (14.2)
Unable	55 (21.7)
Missing	1 (0.4)
Usual activities (e.g., work, study, housework, family or leisure activities)	
No problems	44 (17.3)
Slight problems	58 (22.8)
Moderate problems	65 (25.6)
Severe problems	42 (16.5)
Unable	44 (17.3)
Missing	1 (0.4)
Pain / discomfort	
No pain or discomfort	121 (47.6)
Slight pain or discomfort	76 (29.9)
Moderate pain or discomfort	40 (15.7)
Severe pain or discomfort	11 (4.3)
Extreme pain or discomfort	5 (2.0)
Missing	1 (0.4)
Anxiety / depression	
Not anxious or depressed	123 (48.4)
Slightly anxious or depressed	89 (35.0)
Moderately anxious or depressed	27 (10.6)
Severely anxious or depressed	8 (3.1)
Extremely anxious or depressed	6 (2.4)
Missing	1 (0.4)
EQ-VAS, *n*	253
Mean±SD	80.19 ±15.57
EQ-5D-5L index score, *n*	253
Mean±SD	0.56 ±0.24
MMSE, *n*	246
Mean±SD	15.76 ±8.46
MMSE category	
No cognitive impairment: 30	6 (2.4)
Mild cognitive impairment: 26–29	21 (8.3)
Mild dementia: 21–25	54 (21.3)
Moderate dementia: 11–20	105 (41.3)
Severe dementia: 0–10	60 (23.6)
Missing	8 (3.1)
Barthel index, *n*	247
Mean±SD	52.47 ±30.58
Prescribed anti-dementia drugs	
Donepezil	19 (7.5)
Galantamine	5 (2.0)
Rivastigmine	4 (1.6)
Memantine	16 (6.3)
Yokukansan/YiganSan	14 (5.5)

SD, standard deviation; VAS, visual analogue scale, MMSE, Mini-Mental State Examination, Data are presented as frequencies and percentages unless otherwise indicated. Percentages may not sum up to 100% due to rounding.

#### Item analysis

Table 2 shows the distributions of the EQ-5D-5L+C response at three time points. No ceiling or floor effect (>70%) was observed in any of the six dimensions of the EQ-5D-5L+C. Missing data was as small as 1.6% at 3 months at most among three time points.

**Table 2:** Distribution of EQ-5D-5L+C response at baseline, 3 months, and 6 months

EQ-5D-5L+C	Baseline		3 months		6 months	
	*n*	(%)	*n*	(%)	*n*	(%)
Total, *n*	254		254		235	
Mobility						
No problems	45	(17.7)	48	(18.9)	39	(16.6)
Slight problems	55	(21.7)	39	(15.4)	39	(16.6)
Moderate problems	30	(11.8)	25	(9.8)	27	(11.5)
Severe problems	29	(11.4)	43	(16.9)	29	(12.3)
Unable	93	(36.6)	95	(37.4)	99	(42.1)
Missing	2	(0.8)	4	(1.6)	2	(0.9)
Self-care						
No problems	56	(22.0)	52	(20.5)	47	(20.0)
Slight problems	58	(22.8)	56	(22.0)	57	(24.3)
Moderate problems	49	(19.3)	36	(14.2)	31	(13.2)
Severe problems	35	(13.8)	38	(15.0)	33	(14.0)
Unable	55	(21.7)	68	(26.8)	65	(27.7)
Missing	1	(0.4)	4	(1.6)	2	(0.9)
Usual activities						
No problems	44	(17.3)	38	(15.0)	34	(14.5)
Slight problems	60	(23.6)	55	(21.7)	64	(27.2)
Moderate problems	64	(25.2)	56	(22.0)	43	(18.3)
Severe problems	41	(16.1)	55	(21.7)	46	(19.6)
Unable	44	(17.3)	46	(18.1)	46	(19.6)
Missing	1	(0.4)	4	(1.6)	2	(0.9)
Pain / discomfort						
No pain or discomfort	120	(47.2)	95	(37.4)	93	(39.6)
Slight pain or discomfort	74	(29.1)	98	(38.6)	90	(38.3)
Moderate pain or discomfort	41	(16.1)	35	(13.8)	36	(15.3)
Severe pain or discomfort	12	(4.7)	12	(4.7)	7	(3.0)
Extreme pain or discomfort	6	(2.4)	10	(3.9)	7	(3.0)
Missing	1	(0.4)	4	(1.6)	2	(0.9)
Anxiety / depression						
Not anxious or depressed	118	(46.5)	128	(50.4)	105	(44.7)
Slightly anxious or depressed	90	(35.4)	83	(32.7)	89	(37.9)
Moderately anxious or depressed	30	(11.8)	26	(10.2)	21	(8.9)
Severely anxious or depressed	8	(3.1)	7	(2.8)	14	(6.0)
Extremely anxious or depressed	7	(2.8)	6	(2.4)	4	(1.7)
Missing	1	(0.4)	4	(1.6)	2	(0.9)
Cognition						
No problems	32	(12.6)	44	(17.3)	31	(13.2)
Slight problems	90	(35.4)	70	(27.6)	67	(28.5)
Moderate problems	73	(28.7)	75	(29.5)	80	(34.0)
Severe problems	39	(15.4)	46	(18.1)	44	(18.7)
Extreme problems	17	(6.7)	15	(5.9)	11	(4.7)
Missing	3	(1.2)	4	(1.6)	2	(0.9)
EQ-VAS						
Missing	1		4		2	
Mean±SD	80.2	±15.56	73.03	±14.64	75.5	±15.26

EQ-5D-5L+C, cognition bolt-on version of the Japanese EQ-5D, VAS, visual analogue scale. Percentages may not sum up to 100% due to rounding.

#### Validity

For construct validity, the strongest correlation (95% confidence interval, CI) with the MMSE score was found in the cognition dimension (*r*_s_ = – 0.640 [CI: – 0.730 to – 0.550]), followed by a strong and moderate correlation in the self-care and usual activities dimensions, respectively (*r*_s_ = – 0.530 [– 0.632 to – 0.429] and – 0.497 [– 0.598 to – 0.395], respectively) (Table 3).

**Table 3:** Correlations between EQ-5D-5L+C dimensions and MMSE at baseline

EQ-5D-5L+C dimensions	*n*	Spearman’s correlation coefficient	(95% CI)
Mobility	245	– 0.330	(– 0.447 to – 0.213)
Self-care	246	– 0.530	(– 0.632 to – 0.429)
Usual activities (e.g., work, study, housework, family, or leisure activities)	246	– 0.497	(– 0.598 to – 0.395)
Pain / discomfort	246	– 0.026	(– 0.153 to 0.102)
Anxiety / depression	246	– 0.033	(– 0.161 to 0.094)
Cognition (memory, comprehension, concentration, thinking)	244	– 0.640	(– 0.730 to – 0.550)

EQ-5D-5L+C, cognition bolt-on version of the Japanese EQ-5D, MMSE, Mini-Mental State Examination, CI, confidence interval. The number of participants varied due to missing data among each dimension.

#### Reliability

In a total of 138 and 119 participants, the MMSE category was unchanged at 3 months and 6 months from baseline, respectively. Of these participants, a good level of agreement was observed both between baseline and 3 months and between baseline and 6 months (*k* = 0.644, 95% CI, 0.541 to 0.746; and *k* = 0.656, 0.549 to 0.763, respectively) (Table 4).

**Table 4:** Agreement of the cognition dimension between baseline and 3 months and between baseline and 6 months among participants whose MMSE categories were unchanged from baseline

	Response at 3 months (*n* = 138), *n* (%)					Weighted	(95% CI)
Baseline response	No problems	Slight problems	Moderate problems	Severe problems	Extreme problems	kappa coefficient^*^
No problems	9 (6.5)	1 (0.7)	2 (1.4)	0 (0.0)	0 (0.0)	0.644	(0.541 to 0.746)
Slight problems	8 (5.8)	20 (14.5)	20 (14.5)	2 (1.4)	0 (0.0)		
Moderate problems	1 (0.7)	5 (3.6)	17 (12.3)	7 (5.1)	2 (1.4)		
Severe problems	2 (1.4)	3 (2.2)	2 (1.4)	13 (9.4)	8 (5.8)		
Extreme problems	0 (0.0)	1 (0.7)	2 (1.4)	10 (7.2)	3 (2.2)		
	Response at 6 months (*n* = 119), *n* (%)				
No problems	7 (5.9)	3 (2.5)	0 (0.0)	0 (0.0)	0 (0.0)	0.656	(0.549 to 0.763)
Slight problems	1 (0.8)	19 (16.0)	17 (14.3)	5 (4.2)	0 (0.0)		
Moderate problems	1 (0.8)	9 (7.6)	19 (16.0)	5 (4.2)	0 (0.0)		
Severe problems	0 (0.0)	2 (1.7)	9 (7.6)	9 (7.6)	3 (2.5)		
Extreme problems	0 (0.0)	0 (0.0)	1 (0.8)	6 (5.0)	3 (2.5)		

MMSE, Mini-Mental State Examination. ^ *^Fleiss-Cohen weights were used.

#### Sensitivity to change

The mean MMSE score changed by – 1.207 (SD: 5.346) at 3 months and by – 1.149 (5.899) at 6 months from baseline among 237 and 221 participants, respectively (Table 5). A similar but weak correlation between changes in the cognition dimension and those in the MMSE score was observed for both two sets of time points (3 months: *r*_s_ = – 0.191, 95% CI, – 0.325 to – 0.058; 6 months: *r*_s_ = – 0.267, – 0.396 to – 0.138). Although weak, a correlation with changes in the MMSE score at 3 months was higher when the cognition dimension was added to the EQ-5D-5L compared to the EQ-5D alone (*r*_s_ = – 0.142, – 0.275 to – 0.009; versus *r*_s_ = – 0.074, – 0.207 to 0.059) (Table 6).

**Table 5:** Sensitivity to MMSE change in the cognition dimension

	3 months versus baseline	6 months versus baseline
*n*	237	221
Difference in EQ-5D-5L+C cognition level, Mean±SD	0.017±0.916	0.140±0.945
Difference in MMSE, Mean±SD	– 1.207±5.346	– 1.149±5.899
Worsening in EQ-5D-5L+C cognition, %	29.1	33.9
Worsening in MMSE, %	53.6	48.4
Spearman correlation coefficient (95% CI)	– 0.191 (– 0.325 to – 0.058)	– 0.267 (– 0.396 to – 0.138)

EQ-5D-5L+C, cognition bolt-on version of the Japanese EQ-5D, SD, standard deviation, MMSE, Mini-Mental State Examination, CI, confidence interval.

**Table 6:** Sensitivity to MMSE change in EQ-5D-5L (5 dimensions) and EQ-5D-5L+C (6 dimensions)

	Total levels (1– 5) of Sum of EQ-5D-5L dimensions at 3 months	Total levels (1– 5) of Sum of EQ-5D-5L+C 6 dimensions at 3 months
*n*	239	236
Difference in the corresponding score, mean±SD	0.464±3.175	0.395±3.140
Difference in MMSE, mean±SD	– 1.238±5.338	– 1.208±5.357
Worsening in the corresponding score, %	43.9	45.3
Worsening in MMSE, %	54.0	53.4
Spearman correlation coefficient (95% CI)	– 0.074 (– 0.207 to 0.059)	– 0.142 (– 0.275 to – 0.009)

EQ-5D-5L+C, cognition bolt-on version of the Japanese EQ-5D, MMSE, Mini-Mental State Examination, CI, confidence interval.

## DISCUSSION

This study aimed to develop the EQ-5D-5L+C capturing health status in individuals with cognitive impairment among Japanese by adding the cognition dimension to the Japanese EQ-5D-5L. To achieve this, we conducted linguistic validation for the added cognition dimension, followed by the psychometric validation of the Japanese EQ-5D-5L+C. As a result, we found that the Japanese proxy version of the EQ-5D-5L+C is a valid tool to capture health status including cognitive function in the elderly nursing home residents in Japan with regards to validity and reliability. A careful consideration may, however, be needed to capture cognitive changes.

At the first phase of this study, the Japanese EQ-5D-5L was modified by adding the cognition dimension to it with reference to the bolt-on version previously published by Janssen et al. [6] and validating the cognition dimension linguistically after the permission was granted by EuroQol. A cross-cultural adaptation was performed based on published guidelines [12, 13]. Through the adaptation process, the added cognition dimension in Japanese is conceptually equivalent to the original version with understandability and acceptability, considering for Japanese-speaking individuals.

Our results of construct validity were consistent with the hypotheses generated by previous studies in patients with cognitive impairment [3, 24]. Although the expression of the dimension, assessment time points, and levels in response were not identical, our results were overall consistent with previous findings [3], with an exception of our moderate correlation in the mobility dimension in this study. Our results appeared to be reasonable given that the degree of self-care and usual activities can be affected by one’s cognitive function while the MMSE intends to measure cognitive function. These infers that self-care and usual activities dimensions capture one’s cognitive function to some degree in the EQ-5D-5L, but adding the cognition dimension where cognitive decline is inevitable with aging benefits in capturing cognitive function directly to assess one’s health state.

A good level of test-retest reliability of the cognitive dimension was observed in our results. Given that the data was collected during routine work of proxies who are caregivers at the nursing homes and that the data was collected during the COVID-19 pandemic, feasibility of this study was prioritized and assessments by identical proxies in each participant among all assessment points were compromised. This suggests that multiple proxies who engage in caregiving for the target for evaluation can make a sufficiently consistent assessment using the proxy version of the EQ-5D-5L+C.

The results in sensitivity to change of the cognition dimension in relation to change in the MMSE score were as weak as the previous study [3]. This may explain differences in ability to capture changes in cognitive impairment over time by the MMSE and cognitive dimension due to the difference in scaling. While the MMSE is tested on the target for evaluation based on the 11 items covering five areas of cognitive function, with a wide range of score from 0 to 30, the cognition dimension is one item rated on a five-level scale. The mean changes in the MMSE score appeared to be virtually unchanged at 3 months and 6 months (– 1.207±5.346 and – 1.149±5.899, respectively) despite the score range. When we plotted the changes in the MMSE score according to the changes in the cognition dimension at 3 months and 6 months, participants with greater deterioration in the cognition dimension tend to have greater decline in MMSE particularly at 6 months (data not shown). Longer assessment period may be needed to ensure that the EQ-5D-5L+C captures changes in cognitive impairment based on the MMSE score or other criteria. Although weak, our results indicate that adding the cognition dimension to the EQ-5D-5L captures the changes in cognitive impairment which the MMSE apprehends, suggesting the benefits in assessing health state using this cognition bolt-on version. However, a careful consideration is needed in use to capture cognitive changes.

### Limitations

The results of this study need to be interpreted with care. First, as the participants of the pilot test in the linguistic validation were recruited from online panel who had been voluntarily registered in advance, they may have been more motivated and familiar to participate in surveys or studies in general. Selection bias may not be inevitable because they are not necessarily representative of caregivers in general. Moreover, the participants were limited to caregivers of individuals with cognitive impairment in this pilot test.

Second, the diagnosis data from medical institutions were not available in this research setting. This prevented us from identifying those with delirium or acute illness who may have experienced high variability and also led us to classify those with dementia or mild cognitive impairment based solely on the MMSE scores which was developed for screening purposes and assessed on a regular basis at the nursing homes. Since there are no agreed-upon cut offs for the MMSE scores, we have employed the widely used thresholds proposed by Perneczky et al. [21] where the MMSE scores can be used as a surrogate measure for staging dementia based on the Clinical Dementia Rating (CDR). For future studies, it is desirable to collect the diagnosis data to see if any misclassification of the participants exist.

Third, the nursing home residents and their proxies do not necessarily represent the elderly with various degrees of cognitive impairment and their caregivers. Our participants did not include the elderly who visit care facilities on a regular basis or who stay and receive care at home. In addition, the proxies in this study had prior experience in answering questionnaires including the EQ-5D-5L for their residents. They may have been somewhat familiar with responding to the EQ-5D-5L+C unlike perhaps most caregivers at home. Further psychometric properties of the EQ-5D-5L+C may be needed for individuals who receive care at home formally or informally.

Next, it should be noted that proxy responses may not necessarily match with the resident’s responses as previously reported [24, 28]. Reasonable discrepancies in responses between proxies and participants are inevitable. Careful interpretation of the results may be needed.

Lastly, we developed the proxy version of the EQ-5D-5L+C in parallel with its self-complete version. Linguistic validation and psychometric properties of the self-complete versions are not yet assessed. The difference between those two versions, however, exists only in the instructions specifying the target for evaluation. Regardless of all these limitations, our study is unique in validating and developing the EQ-5D-5L+C among the elderly individuals in the Japanese aging society.

### Conclusion

The Japanese proxy version of the EQ-5D-5L+C was developed through linguistic and psychometric validations in this study. The EQ-5D-5L+C is a valid tool to capture health status including the cognition in the elderly nursing home residents in Japan, confirming the understandability, good psychometric properties in validity and reliability. A careful consideration may be needed to assess cognitive changes over time, yet the benefits in adding the cognition dimension to the EQ-5D-5L to assess health state was suggested. Future research is needed to develop value sets for the EQ-5D-5L+C.

## Supplementary Material

Supplementary Material

## Data Availability

The data supporting the findings of this study are not publicly available due to privacy and ethical reasons.
