# Supplementary material for: Linguistic and Psychometric Validation of the Cognition Bolt-On Version of the Japanese EQ-5D-5L for the Elderly
Source: J Alzheimers Dis. 2023 Feb 14;91(4):1447–58. doi: 10.3233/JAD-221080 (PMC9986695; doi:10.3233/JAD-221080)
Supplement: Supplementary Material [file jad-91-jad221080-s001.pdf]

Supplementary Material

Linguistic and Psychometric Validation of the Cognition Bolt-On Version of the Japanese EQ-5D-5L for the Elderly

Supplementary Figure 1. Finalized Japanese cognition dimension

認知機能 *（例：記憶力[もの忘れ]、理解力、集中力、思考力）*

認知機能に問題はない	❑
認知機能に少し問題がある	❑
認知機能に中程度の問題がある	❑
認知機能にかなり問題がある	❑
認知機能に極度の問題がある	❑

© EuroQol Research Foundation. EQ-5D™ is a trademark of the EuroQol Research Foundation.

Reproduced by permission of EuroQol Research Foundation. This is a modified EQ-5D.

Reproduction of this version is not allowed. For reproduction, use or modification of the EQ-5D (any version), please register your study by using the online EQ registration page: www.euroqol.org.
